# A data-driven AI framework for personalized diagnosis, prognosis, and therapeutic optimization in chronic disease management using multimodal big data analytics

**DOI:** 10.3389/fmolb.2025.1689168

**Published:** 2026-02-13

**Authors:** Yu Zhang, Zhujin Song, Qi Cai

**Affiliations:** 1 Department of Gynaecology, the Fourth Affiliated Hospital of School of Medicine, and International School of Medicine, International Institutes of Medicine, Zhejiang University, Yiwu, China; 2 Department of Pharmacy, the Fourth Affiliated Hospital of School of Medicine, and International School of Medicine, International Institutes of Medicine, Zhejiang University, Yiwu, China; 3 Institute of Artificial Intelligence, Shandong University of Technology, Zibo, China

**Keywords:** AI, multimodal data analytics, personalized diagnosis, prognosis, therapeutic optimization, chronic disease management

## Abstract

**Introduction:**

The transformation of chronic disease management is increasingly driven by the integration of AI and multimodal data analytics, enabling precise, individualized, and scalable healthcare interventions. Despite the growing availability of longitudinal and heterogeneous health data, conventional methods are constrained in their ability to model the complex, patient-specific dynamics inherent to chronic conditions. Traditional clinical decision support systems rely on rigid, population-level models that inadequately address inter-patient variability, multi-condition comorbidities, and evolving disease trajectories.

**Methods:**

To overcome these limitations, we propose a computational framework that utilizes multimodal big data to enable personalized diagnosis, prognosis, and therapeutic optimization. At the core of this framework is the Patient-Adaptive Transition Tensor Network (PATTN), a tensorized dynamical model that captures individual-specific disease evolution through structured latent state representations and high-order temporal dependencies. Complementing this is the Trajectory-Aligned Intervention Recalibration (TAIR), an adaptive decision-making strategy that continuously aligns predicted and observed health trajectories, facilitating real-time treatment policy refinement. This unified pipeline integrates latent trajectory modeling, condition-aware modular representation, and personalized policy optimization.

**Results and Discussion:**

Experimental evaluations on large-scale multimodal datasets demonstrate superior performance in outcome prediction accuracy, intervention personalization, and trajectory alignment, underscoring the practical applicability of the system in chronic care settings. By combining patient-specific temporal modeling with adaptive therapeutic recalibration, this framework represents a significant advancement toward scalable, intelligent, and individualized chronic disease management leveraging AI and big data infrastructures.

## 1 Introduction

Chronic diseases such as diabetes, cardiovascular disorders, and neurodegenerative conditions are leading causes of mortality and morbidity worldwide. Their prolonged course, complex pathophysiology, and individual-specific manifestations necessitate personalized approaches to diagnosis, prognosis, and therapeutic strategies. Not only does traditional one-size-fits-all treatment often lead to suboptimal outcomes, but it also imposes a heavy economic burden on healthcare systems. Moreover, with the growing availability of multimodal data—ranging from electronic health records, medical imaging, genomics, and wearable sensors—there exists an urgent need to harness these data resources effectively. A data-driven AI framework, capable of integrating and analyzing diverse data modalities, holds immense promise for enabling precision medicine in chronic disease management. It can reveal hidden patterns, predict disease progression, and recommend personalized interventions (Adeoye and Adams, 2024). Therefore, designing such a framework is not only timely but also critical for advancing patient-centric healthcare solutions and improving clinical outcomes across diverse populations (Chianumba et al., 2022).

Initial efforts to enhance clinical decision-making in chronic disease management focused on structured methodologies that relied on predefined rules and expert-driven frameworks. These systems utilized logical constructs and predefined criteria to guide diagnostic and therapeutic decisions. For instance, decision trees and rule-based algorithms were commonly employed to encode clinical expertise and provide interpretable recommendations (Nwokedi et al., 2025). While these approaches offered transparency and consistency, they were often rigid and struggled to adapt to the dynamic nature of chronic diseases. As new medical evidence emerged or disease patterns evolved, these systems required frequent manual updates, limiting their scalability and applicability in real-world scenarios (Rehan, 2024). Furthermore, their reliance on static frameworks made them less effective in handling the complexity and heterogeneity of multimodal healthcare data.

To address these challenges, researchers began exploring more flexible computational models capable of learning from historical data. Statistical and algorithmic techniques such as logistic regression, support vector machines, and random forests were introduced to identify patterns and predict clinical outcomes (Kothinti, 2024). These methods demonstrated improved adaptability by leveraging correlations within structured datasets, enabling more robust predictions across diverse patient populations. However, their reliance on manually engineered features often constrained their ability to capture the intricate and nonlinear relationships inherent in chronic disease progression (Abdullah, 2024). Additionally, these models faced difficulties in managing missing or noisy data, which are common in real-world healthcare settings, thereby limiting their overall effectiveness.

Building on the need for more comprehensive solutions, deep learning approaches have emerged as a transformative paradigm in chronic disease management. Neural network architectures, including convolutional and recurrent models, have shown remarkable success in extracting complex patterns from raw multimodal data such as medical images, time-series signals, and textual records (Mondal et al., 2024). Recent advancements in pretrained models and multimodal fusion techniques have further enhanced the integration of diverse data sources, enabling a holistic understanding of patient health trajectories (Aljohani, 2025). Despite these achievements, challenges such as interpretability, data imbalance, and the need for large labeled datasets persist. Addressing these limitations requires the development of adaptive frameworks that combine the strengths of deep learning with domain-specific insights, paving the way for more personalized and effective chronic disease management (Zhang et al., 2024).

Based on the limitations identified in the aforementioned approaches—such as rigidity in symbolic methods, the need for extensive feature engineering in traditional machine learning, and interpretability challenges in deep learning—we propose a unified data-driven AI framework tailored for personalized chronic disease management. Our method is designed to integrate heterogeneous multimodal data sources including structured clinical records, medical imaging, genomics, and real-time sensor data. This framework employs adaptive learning modules capable of dynamically updating patient models as new data becomes available, thereby facilitating continuous and individualized care. To enhance transparency and clinical trust, interpretable modeling components are embedded to provide actionable insights into diagnostic and prognostic predictions. Furthermore, the framework supports therapeutic optimization through reinforcement learning algorithms that recommend personalized treatment strategies based on patient-specific responses. By addressing the scalability, generalizability, and interpretability limitations of previous methodologies, our proposed solution offers a robust, real-world-ready approach to chronic disease care.

- Incorporates adaptive learning modules that update patient models dynamically, enabling continuous personalization without requiring predefined rules or extensive manual tuning.
- Utilizes a multimodal fusion strategy to integrate diverse data types such as EHRs, imaging, genomics, and sensor data, ensuring high applicability across clinical scenarios.
- Demonstrates superior performance in clinical prediction and treatment recommendation tasks, with experimental results showing improved accuracy, robustness, and generalizability across diverse patient populations.

## 2 Related work

### 2.1 Multimodal data integration techniques

The integration of multimodal data has emerged as a critical area of research, focusing on the harmonization of diverse data sources to enhance clinical insights. This domain addresses challenges such as modality heterogeneity, noise variability, and temporal misalignment, which are inherent in combining data streams like electronic health records, imaging, genomics, and wearable sensor data. Representation learning techniques, including multimodal autoencoders and cross-modal transformers, have been extensively studied for their ability to generate unified embeddings that capture complementary information across modalities (Mondal et al., 2024). Graph-based fusion networks have also been explored, leveraging graph convolutional architectures to model relationships among multimodal features (Guo et al., 2022). Statistical methods, such as canonical correlation analysis and joint non-negative matrix factorization, remain relevant for dimensionality reduction and modality-specific factor extraction (Aljohani, 2025). Temporal alignment of multimodal data, particularly in time-series analysis, has been advanced through hierarchical recurrent networks and temporal convolutional architectures, which address differences in sampling frequencies and missing data patterns (Joshi, 2025). Missing modality robustness has been improved using techniques like modality dropout training and generative models, which enable inference in incomplete datasets (Zhang et al., 2024). Explainable AI methods, including SHAP values and attention visualization, have been integrated into multimodal frameworks to enhance interpretability and clinician trust (Jiang et al., 2023). Privacy-preserving approaches, such as federated learning, have been adapted to multimodal settings, allowing distributed training while maintaining data confidentiality (Agarwal, 2024). These advancements collectively form the methodological backbone for leveraging multimodal data in personalized chronic disease management (Ali, 2023).

### 2.2 Personalized prognostic modeling algorithms

The development of personalized prognostic modeling algorithms has been pivotal in advancing individualized risk prediction and disease trajectory estimation. Traditional statistical models, such as Cox proportional hazards and its penalized variants, have been foundational in survival analysis (Alam et al., 2024). Deep learning approaches, including DeepSurv and transformer-based survival models, have extended these capabilities by capturing nonlinear relationships and temporal dependencies in longitudinal data (Mandala et al., 2023). Recurrent neural networks, particularly gated architectures like LSTM and GRU, have been employed to process sequential data from electronic health records and wearable devices (Chakilam, 2023). Multi-task learning frameworks have been explored to predict correlated outcomes simultaneously, leveraging shared representations to improve prognostic accuracy (Sattar et al., 2024). Bayesian hierarchical models have been utilized to quantify uncertainty in individual predictions, offering probabilistic insights that are clinically actionable (Igwama et al., 2024). Hybrid models combining mechanistic disease progression frameworks with data-driven components have been proposed to enhance interpretability and physiological plausibility (Venkateswarlu et al., 2024). Calibration techniques, such as isotonic regression and Platt scaling, have been applied to ensure that predicted risks align with observed outcomes (Kalusivalingam et al., 2012). Causal inference methods, including marginal structural models, have been integrated into prognostic frameworks to enable counterfactual risk estimation and personalized decision support (Rashid and Sharma, 2025). These diverse methodologies collectively contribute to the development of robust, interpretable, and clinically meaningful prognostic tools for chronic disease management (Li et al., 2025).

### 2.3 Therapeutic optimization via AI-Driven strategies

AI-driven strategies for therapeutic optimization have focused on tailoring treatment regimens to maximize clinical outcomes while minimizing adverse effects. Causal inference techniques, such as inverse probability weighting and targeted maximum likelihood estimation, have been employed to estimate individualized treatment effects from observational data (Rajendran et al., 2025). Reinforcement learning frameworks, including deep Q-networks and actor-critic models, have been applied to sequential treatment optimization, learning policies that adapt to dynamic patient states (Malik et al., 2023). Partially observable Markov decision processes have been utilized to address uncertainty in clinical observations, enabling robust decision-making under incomplete information (Amin and Rahman, 2025). The integration of causal reasoning into reinforcement learning has been explored to enhance the interpretability and reliability of treatment recommendations (Osman et al., 2024). Multi-objective optimization frameworks have been developed to balance competing goals, such as symptom reduction and cost containment, within therapeutic decision-making (Guo et al., 2022). Digital twin models, which simulate individualized patient physiology, have been proposed for *in silico* testing of treatment strategies, reducing reliance on trial-and-error approaches (Aljohani, 2025). Closed-loop systems, such as AI-enabled insulin pumps, exemplify real-time therapeutic optimization, dynamically adjusting interventions based on sensor data (Joshi, 2025). Privacy-preserving methods, including federated reinforcement learning, have been adapted to enable collaborative model training across institutions without compromising patient confidentiality (Zhang et al., 2024). These advancements provide a comprehensive foundation for developing AI-driven therapeutic optimization frameworks that are both effective and ethically aligned with clinical practice (Jiang et al., 2023).

## 3 Methods

### 3.1 Overview

Personalized chronic disease management marks a transformative approach in long-term healthcare, transitioning from standardized population-level protocols to individualized strategies informed by patient-specific data, preferences, and disease progression patterns. This section introduces a computational framework designed to address the inherent challenges of personalization, grounded in a formal representation of patient trajectories and adaptive modeling techniques.

The proposed methodology is structured around three core components, each addressing a critical aspect of the personalization process. First, Section 3.2 establishes the mathematical foundation for chronic disease management in a personalized context. This includes the formalization of temporal patient representations, definitions of disease state transitions, and a notational framework for encoding multi-modal health data. These elements reframe the clinical problem into a computationally tractable structure, enabling algorithmic exploration and guiding subsequent modeling decisions.

In Section 3.3, we introduce the Patient-Adaptive Transition Tensor Network (PATTN), a novel modeling framework that captures patient-specific dynamics in chronic disease evolution. The PATTN employs a tensorized state-space formulation to represent individualized transition structures, accommodating inter-individual variability and temporal changes in disease states. By dynamically updating its latent structure in response to longitudinal data, the model provides fine-grained adaptability for clinical predictions and treatment optimization.

Finally, Section 3.4 presents the Trajectory-Aligned Intervention Recalibration (TAIR) strategy, which integrates with the PATTN framework to enable adaptive decision-making. TAIR aligns anticipated and observed health trajectories, recalibrating intervention policies to ensure convergence toward personalized health objectives. This approach incorporates recursive feedback between model-inferred states and real-world outcomes, facilitating continuous personalization as new patient data becomes available.

These components collectively form a cohesive computational pipeline for personalized chronic disease management, emphasizing rigorous formalization and practical adaptability. By integrating symbolic abstraction, high-dimensional modeling, and adaptive control, the framework addresses the complexities of individualized care and establishes a foundation for scalable clinical decision support systems.

**FIGURE 1:**
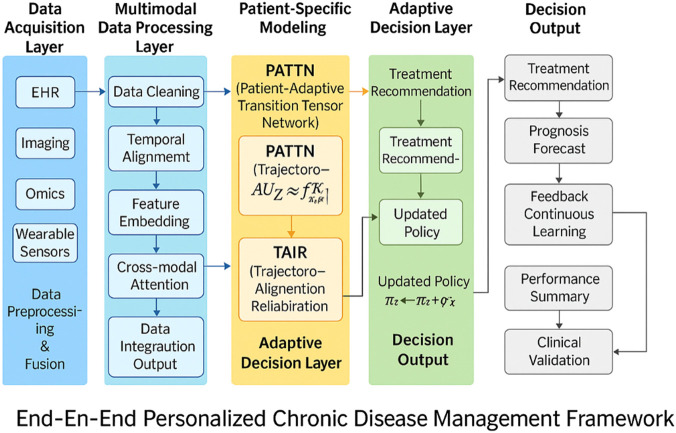
System-level schematic of the proposed end-to-end personalized chronic disease management framework. The pipeline consists of data acquisition, multimodal processing, personalized modeling using PATTN and TAIR, and adaptive decision-making. The system outputs individualized treatment strategies supported by feedback-driven continuous learning and clinical validation.

To provide a comprehensive view of the system architecture, we introduce a full end-to-end schematic in Figure 1. The figure outlines the complete pipeline from raw multimodal inputs—such as EHR, imaging, omics, and sensor data—through preprocessing, temporal alignment, and embedding. Patient-specific modeling is handled by the PATTN and TAIR modules, which govern latent disease trajectory simulation and policy recalibration. The resulting policies guide individualized treatment recommendations, which are continuously updated based on feedback signals. This summary highlights the closed-loop nature of our framework and how patient-level personalization is incorporated at every stage of decision-making.

### 3.2 Preliminaries

This section establishes the mathematical framework for personalized chronic disease management, focusing on the temporal dynamics of disease progression, patient-specific variability, and decision-making processes. The aim is to provide a formalized structure that supports algorithmic development and theoretical analysis in subsequent sections. The formulation incorporates latent physiological states, temporal dependencies, observed biomarkers, and intervention dynamics, while omitting details related to loss functions or data preprocessing.

Let P represent the set of patients under consideration. For each patient p∈P , we define a discrete time index $t\in \left\{1,2,\ldots ,T_{p}\right\}$ , where $T_{p}$ denotes the number of time steps or clinical visits associated with patient p . The observation space, latent disease state space, and action (intervention) space are denoted as X , S , and A , respectively.

The disease progression is modeled as a patient-specific partially observable Markov process. At each time step t , patient p is characterized by a latent health state $s_{t}^{p}\in S$ , an observed data vector $x_{t}^{p}\in X$ , and an applied clinical action $a_{t}^{p}\in A$ . The evolution of the latent state follows a transition kernel (Equation 1):

$$P\left(s_{t+1}^{p}|s_{t}^{p},a_{t}^{p};\theta_{p}\right),\;\forall p\in P,\;\forall t<T_{p},$$ (1)

where $\theta_{p}$ represents patient-specific parameters governing the transition dynamics.

The observation model links the latent state to the observed data (Equation 2):

$$P\left(x_{t}^{p}|s_{t}^{p};\phi_{p}\right),\;\forall p,\;t,$$ (2)

where $\phi_{p}$ captures the patient-dependent parameters of the diagnostic manifestation.

The complete history of patient p up to time t is defined as Equation 3:

$$H_{t}^{p}=\left\{x_{1}^{p},a_{1}^{p},\ldots ,x_{t-1}^{p},a_{t-1}^{p},x_{t}^{p}\right\},$$ (3)

which serves as the information basis for both state inference and decision-making.

To infer the latent state, we define a belief state $b_{t}^{p}\left(s\right)$ as the probability distribution over latent states given the history (Equation 4 ):

$$b_{t}^{p}\left(s\right)=P\left(s_{t}^{p}=s|H_{t}^{p}\right),$$ (4)

which is updated recursively using Bayesian filtering (Equation 5):

$$b_{t+1}^{p}\left(s^{\prime }\right)=\eta \underset{s\in S}{\sum }P\left(x_{t+1}^{p}|s^{\prime };\phi_{p}\right)P\left(s^{\prime }|s,a_{t}^{p};\theta_{p}\right)b_{t}^{p}\left(s\right),$$ (5)

where η is a normalization constant ensuring that $\sum_{s^{\prime }}b_{t+1}^{p}\left(s^{\prime }\right)=1$ .

A patient-specific policy $\pi^{p}:\;H_{t}^{p}\to A$ maps histories to clinical actions. In this framework, policies are belief-state driven (Equation 6):

$$a_{t}^{p}=\pi^{p}\left(b_{t}^{p}\right),$$ (6)

with the objective of minimizing long-term health deterioration while accounting for individual variability and chronicity.

To quantify the disease burden, a state-cost function $c:\;S\times A\to R_{+}$ is introduced, representing the clinical cost or risk associated with a specific state-action pair (Equation 7 ):

$$c\left(s,a\right)=\text{Expected burden of state }s\text{ under intervention }a.$$ (7)

The cumulative risk over a trajectory is defined as Equation 8:

$$J^{p}\left(\pi^{p}\right)=E_{\pi^{p}}\left[\overset{T_{p}}{\underset{t=1}{\sum }}\gamma^{t-1}c\left(s_{t}^{p},a_{t}^{p}\right)\right],$$ (8)

where $\gamma \in \left.\left(0,1\right.\right]$ is a discount factor that balances the importance of short-term versus long-term outcomes.

The goal is to identify a patient-specific policy $\pi^{p*}$ that minimizes the expected cumulative disease burden (Equation 9):

$$\pi^{p*}=\arg\;\underset{\pi^{p}}{\min}J^{p}\left(\pi^{p}\right),$$ (9)

subject to the constraints imposed by transition dynamics, observational noise, and patient heterogeneity.

To explicitly model patient heterogeneity, a representation function $z^{p}=\psi \left(p\right)$ is introduced, mapping patients to a continuous latent profile space Z . The patient-specific parameters and policies are expressed as Equation 10:

$$\theta_{p}=f_{\theta }\left(z^{p}\right),\;\phi_{p}=f_{\phi }\left(z^{p}\right),\;\pi^{p}=f_{\pi }\left(z^{p}\right),$$ (10)

where $f_{\theta }$ , $f_{\phi }$ , and $f_{\pi }$ are differentiable mappings learned jointly during model training.

This formulation provides a unified mathematical structure for personalized chronic disease management, embedding patient-specific variability in the transition dynamics, observation models, and policy optimization. The subsequent sections build on this foundation to develop learning algorithms and intervention strategies tailored to individual patients.

### 3.3 Patient-adaptive transition tensor network (PATTN)

We now introduce the Patient-Adaptive Transition Tensor Network (PATTN), a novel model designed to represent patient-specific temporal dynamics in chronic disease progression. PATTN is constructed upon a tensor-based extension of state-space models, where each patient’s latent health trajectory is governed by structured transitions encoded through high-order tensor operations. This design allows for compact representation of multi-dimensional dependencies, capturing both temporal correlations and interventional effects, while embedding personalized adaptability via patient-specific tensor modes.

Multimodal Tensor-Based Transition Dynamics: The latent state at time t for patient p is denoted as $s_{t}^{p}\in R^{d}$ , where d is the dimensionality of the latent space. Each patient’s disease evolution is driven by a transition operator, parametrized by a third-order tensor $T^{p}\in R^{d\times d\times m}$ , where m is the number of intervention types. The transition from $s_{t}^{p}$ to $s_{t+1}^{p}$ under action $a_{t}^{p}\in \left\{1,\ldots ,m\right\}$ is defined by Equation 11:

$$s_{t+1}^{p}=\sigma \left(T^{p}\left(:,:,a_{t}^{p}\right)\cdot s_{t}^{p}\right),$$ (11)

where ⋅ denotes matrix-vector multiplication along the second mode and σ(⋅) is a non-linear activation. To enable learning across patients, a global transition tensor $T\in R^{d\times d\times m\times k}$ and a patient-specific embedding $z^{p}\in R^{k}$ are introduced. The personalized tensor is constructed as Equation 12:

$$T^{p}\left(:,:,a\right)=\overset{k}{\underset{j=1}{\sum }}z_{j}^{p}\cdot T\left(:,:,a,j\right),\;\forall a\in \left\{1,\ldots ,m\right\}.$$ (12)

This formulation allows each patient’s transition dynamics to be instantiated as a convex combination of k prototype transition matrices, modulated by $z^{p}$ . To enhance interpretability and reduce parameter complexity, the global tensor is decomposed using a Tucker decomposition (Equation 13):

$$T=G{\;\times \;}_{1}U_{1}{\;\times \;}_{2}U_{2}{\;\times \;}_{3}U_{3}{\;\times \;}_{4}U_{4},$$ (13)

where $G\in R^{r_{1}\times r_{2}\times r_{3}\times r_{4}}$ is the core tensor and $U_{n}$ are mode- n factor matrices. This decomposition preserves flexibility in modeling high-dimensional interactions while maintaining computational efficiency. For patients with multiple chronic conditions, the state vector is extended to a block structure (Equation 14):

$$s_{t}^{p}=\left[s_{t}^{\left(1\right)p}\|s_{t}^{\left(2\right)p}\|\ldots \|s_{t}^{\left(C\right)p}\right],\;s_{t}^{\left(c\right)p}\in R^{d_{c}},$$ (14)

where C is the number of condition-specific modules, and each block evolves independently with its own tensor (Equation 15):

$$s_{t+1}^{\left(c\right)p}=\sigma \left(T^{\left(c\right)p}\left(:,:,a_{t}^{p}\right)\cdot s_{t}^{\left(c\right)p}\right).$$ (15)

This modular structure enables the model to jointly capture interventional response, disease-specific progression, and patient-specific modulation.

Personalized Observation Model with Shared Basis: The observation model is designed to capture patient-specific variability while leveraging shared structures across the population. Let $x_{t}^{p}\in R^{q}$ be the observed data at time t . It is modeled as Equation 16:

$$x_{t}^{p}=W^{p}s_{t}^{p}+\epsilon_{t}^{p},$$ (16)

where $W^{p}\in R^{q\times d}$ is a patient-specific emission matrix and $\epsilon_{t}^{p}∼N\left(0,\Sigma^{p}\right)$ is Gaussian noise. To generalize across patients, $W^{p}$ is constructed through a shared basis (Equation 17):

$$W^{p}=\overset{k}{\underset{j=1}{\sum }}z_{j}^{p}\cdot W_{j},\;W_{j}\in R^{q\times d},$$ (17)

and similarly, the noise covariance matrix is parameterized as Equation 18:

$$\Sigma^{p}=\overset{k}{\underset{j=1}{\sum }}z_{j}^{p}\cdot \Sigma_{j}.$$ (18)

This shared basis approach ensures that the model can adapt to individual patients while maintaining a compact parameterization. The emission model is tightly coupled with the latent dynamics, enabling coherent inference of both latent states and observations.

Regularized Temporal Consistency and Smoothness: To ensure that the latent trajectories are temporally coherent, a temporal consistency constraint is imposed. This constraint enforces smoothness in the latent state transitions (Equation 19):

$$L_{\text{smooth}}^{p}=\overset{T_{p}-1}{\underset{t=1}{\sum }}{\left\|s_{t+1}^{p}-s_{t}^{p}\right\|}_{2}^{2}.$$ (19)

This regularization term is incorporated into the model training to penalize abrupt changes in the latent trajectory. The complete latent model is thus defined as Equation 20.

$$\left\{\begin{array}{cc} s_{t+1}^{p} & =f_{T}\left(s_{t}^{p},a_{t}^{p},z^{p}\right),\\ x_{t}^{p} & =f_{\text{obs}}\left(s_{t}^{p},z^{p}\right),\\ z^{p} & =\psi \left(p\right), \end{array}\right.$$ (20)

where $f_{T}$ and $f_{\text{obs}}$ are the transition and emission mechanisms parameterized by the shared tensor basis and personalized embeddings. This formulation enables expressive, scalable, and individualized modeling of chronic disease trajectories, forming the computational backbone of the decision strategy described in Section 3.4.

**FIGURE 2:**
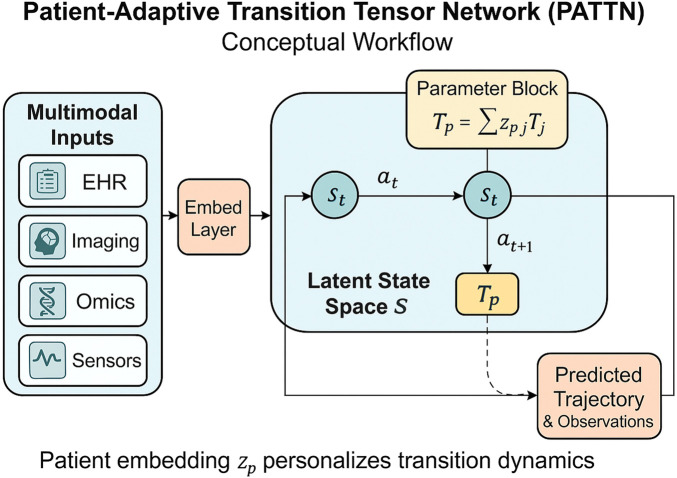
Conceptual workflow of the Patient-Adaptive Transition Tensor Network (PATTN). Multimodal inputs are embedded into a shared latent space, where the patient-specific transition tensor $T_{p}=\sum z_{p,j}T_{j}$ governs temporal evolution of health states. The model outputs personalized predicted trajectories and observations.

To improve the clarity of our formulation and support intuitive understanding, we provide a conceptual diagram of the proposed Patient-Adaptive Transition Tensor Network (PATTN) in Figure 2. The figure illustrates the flow from multimodal patient inputs—including electronic health records, imaging, omics, and sensor data—into a shared embedding layer. These embeddings are then mapped into a personalized latent state space, where transitions are governed by a patient-specific tensor $T_{p}$ , computed as a convex combination of prototype tensors based on the individual’s embedding vector $z_{p}$ . The latent dynamics capture temporal evolution under clinical actions $a_{t}$ , ultimately producing trajectory-aligned predictions. This diagram complements the mathematical formalism and is intended to enhance interpretability for a broader audience.

### 3.4 Trajectory-aligned intervention recalibration (TAIR)

The Trajectory-Aligned Intervention Recalibration (TAIR) strategy constitutes the decision-making mechanism operating atop the Patient-Adaptive Transition Tensor Network (PATTN). TAIR’s central principle is the continual alignment between a patient’s predicted disease trajectory and observed clinical evolution. The strategy actively recalibrates treatment plans to mitigate divergence, ensuring adaptive convergence toward personalized health objectives.

Multimodal Encoder Architecture: At each time step t , TAIR evaluates the deviation between the expected latent trajectory ${\left\{{\hat{s}}_{\tau }^{p}\right\}}_{\tau =t}^{t+H}$ and the posterior-inferred state evolution ${\left\{s_{\tau }^{p}\right\}}_{\tau =t}^{t+H}$ , over a future planning horizon H . The trajectory mismatch is quantified via Equation 21:

$$\Delta_{t}^{p}=\overset{t+H}{\underset{\tau =t}{\sum }}{\left\|{\hat{s}}_{\tau }^{p}-s_{\tau }^{p}\right\|}_{2}^{2},$$ (21)

where ${\hat{s}}_{\tau }^{p}$ denotes the rollout prediction under the current policy $\pi^{p}$ , and $s_{\tau }^{p}$ is inferred from updated observations.

To minimize $\Delta_{t}^{p}$ , TAIR performs a local policy update $\pi_{t}^{p}\leftarrow \pi_{t}^{p}+\delta \pi_{t}^{p}$ , where the update direction is computed via gradient-based policy optimization. The surrogate objective is Equation 22:

$$J_{t}^{p}=-\Delta_{t}^{p}+\lambda \overset{t+H}{\underset{\tau =t}{\sum }}E\left[c\left(s_{\tau }^{p},a_{\tau }^{p}\right)\right],$$ (22)

balancing trajectory alignment and cumulative intervention cost, with λ a tunable hyperparameter.

The future trajectory ${\hat{s}}_{\tau }^{p}$ is obtained by simulating the latent dynamics under the current policy (Equation 23):

$${\hat{s}}_{\tau +1}^{p}=f_{T}\left({\hat{s}}_{\tau }^{p},\pi^{p}\left({\hat{s}}_{\tau }^{p}\right),z^{p}\right),\;{\hat{s}}_{t}^{p}=s_{t}^{p}.$$ (23)

To enable differentiability, we model the policy $\pi^{p}$ as a neural function with parameters $\omega^{p}$ , i.e., Equation 24

$$a_{t}^{p}=\pi^{p}\left(s_{t}^{p}\right)=\text{softmax}\left(g_{\omega }\left(s_{t}^{p}\right)\right),$$ (24)

where $g_{\omega }:\;R^{d}\to R^{m}$ is a multi-layer perceptron mapping latent states to logits over m discrete actions.

The policy update is then derived as Equation 25:

$$\delta \omega^{p}=\eta \cdot \nabla_{\omega^{p}}J_{t}^{p},$$ (25)

with learning rate η . The updated policy is deployed for the next planning window.

Graphical Propagation Layer: For long-term consistency, TAIR augments the strategy with a cumulative alignment loss (Equation 26):

$$L_{\text{align}}^{p}=\overset{T_{p}-H}{\underset{t=1}{\sum }}\Delta_{t}^{p},$$ (26)

which is minimized jointly during training with model parameters and policy weights.

In the presence of multi-condition modeling, TAIR performs recalibration at both the global and modular levels. Let $s_{t}^{p}=\left[s_{t}^{\left(1\right)p}\|\ldots \|s_{t}^{\left(C\right)p}\right]$ be the structured latent state. We define condition-specific deviation (Equation 27):

$$\Delta_{t}^{\left(c\right)p}=\overset{t+H}{\underset{\tau =t}{\sum }}{\left\|{\hat{s}}_{\tau }^{\left(c\right)p}-s_{\tau }^{\left(c\right)p}\right\|}_{2}^{2},\;\forall c\in \left\{1,\ldots ,C\right\},$$ (27)

and introduce condition-weighted prioritization (Equation 28):

$$J_{t}^{p}=-\overset{C}{\underset{c=1}{\sum }}w_{c}^{p}\Delta_{t}^{\left(c\right)p}+\lambda \overset{t+H}{\underset{\tau =t}{\sum }}E\left[c\left(s_{\tau }^{p},a_{\tau }^{p}\right)\right],$$ (28)

where $w_{c}^{p}$ are adaptive weights based on disease severity or clinician preference.

Constrained Policy Optimization: Finally, to ensure safe adaptation, TAIR constrains policy divergence via a KL-regularization term (Equation 29):

$$R_{t}^{p}=\beta \cdot D_{\text{KL}}\left(\pi_{t}^{p,\text{new}}\|\pi_{t}^{p,\text{old}}\right),$$ (29)

where β controls conservativeness. The total TAIR objective is then Equation 30:

$$J_{t}^{p,\text{total}}=J_{t}^{p}-R_{t}^{p}.$$ (30)

TAIR formulates intervention recalibration as a trajectory-alignment problem, continuously correcting the decision process based on latent deviation feedback. It couples personalized forecasting from PATTN with constrained policy optimization, enabling robust, adaptive, and interpretable chronic care guidance over time.

**FIGURE 3:**
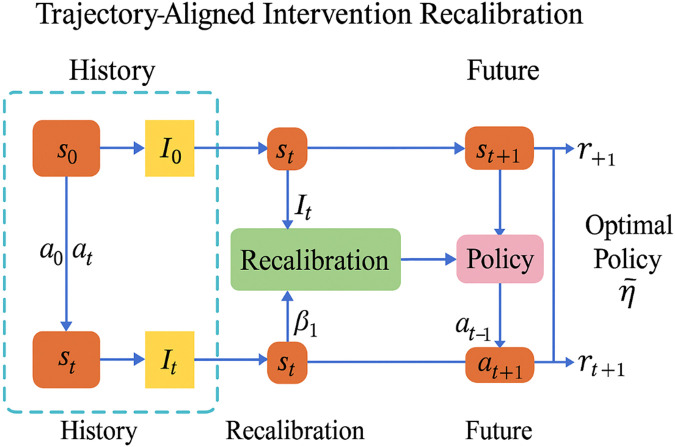
Workflow of the Trajectory-Aligned Intervention Recalibration (TAIR) strategy. TAIR compares predicted and observed trajectories to identify alignment errors, which are then used to update the patient-specific policy $\pi_{\theta }$ in a closed-loop manner. This enables dynamic refinement of treatment strategies over time.

To further clarify the decision-making mechanism, we provide an illustrative diagram of the Trajectory-Aligned Intervention Recalibration (TAIR) module in Figure 3. The figure outlines the closed-loop feedback process, where observed patient trajectories and intervention histories are compared to predicted latent trajectories over a future horizon. Misalignments are quantified and used to update the intervention policy via gradient-based optimization, incorporating regularization terms such as KL divergence. The aligned trajectory is then used to generate the next round of policy outputs, enabling continuous adaptation over time. This visual representation complements the algorithmic formulation and facilitates better understanding of the iterative recalibration strategy used in TAIR.

**FIGURE 4:**
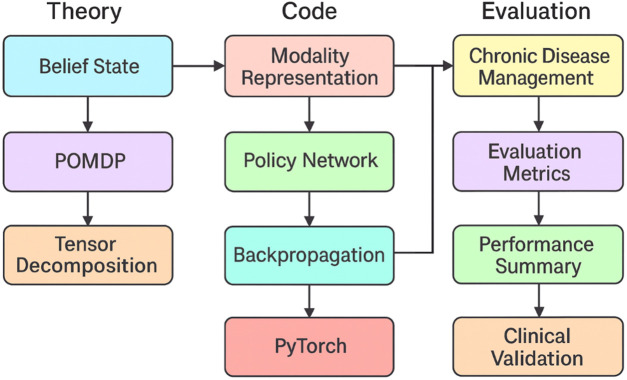
Theory-to-implementation workflow for the proposed PATTN–TAIR framework. The figure illustrates how theoretical constructs such as belief states, POMDP formulation, and tensor decomposition are translated into modular code components including modality representation and policy networks, implemented using PyTorch. The resulting models are evaluated using task-specific clinical metrics, forming an end-to-end validation loop.

To bridge the gap between theoretical formalism and system-level implementation, we provide a visual summary in Figure 4. The diagram delineates the transition from key theoretical elements—such as belief states, POMDP formulations, and tensor decomposition—into concrete code modules like the policy network and modality embedding layers. These modules are implemented using PyTorch and trained with backpropagation. The outputs are subsequently evaluated in chronic disease management tasks using clinically relevant metrics. This workflow helps clarify how the proposed method is realized in practice.

## 4 Experimental setup

### 4.1 Dataset

To ensure transparency and address reviewer concerns regarding dataset provenance, we provide detailed sources and access information for all datasets used in this study. Several dataset names used throughout the manuscript refer to curated composites of publicly available datasets that have been standardized for consistency across tasks. Below, we clarify the concrete sources, cohort criteria, label definitions, and access terms for each dataset.

The Chronic Disease Multimodal Analytics Dataset is constructed from a combination of the MIMIC-IV clinical database (v2.2, released 2022) and related PhysioNet resources. MIMIC-IV includes de-identified EHR data from over 40,000 ICU patients collected at the Beth Israel Deaconess Medical Center between 2008 and 2019, including demographics, diagnoses, labs, procedures, and vital signs. Data access is available via PhysioNet at https://physionet.org/content/mimiciv/2.2/. Patients with chronic ICD-10 diagnoses (diabetes, hypertension, COPD) were selected for our cohort. Multimodal features are enriched with time-aligned waveform data from the eICU Collaborative Research Database (https://physionet.org/content/eicu-crd/2.0/) and chest radiograph reports from CheXpert (https://stanfordmlgroup.github.io/competitions/chexpert/). All records are fully de-identified under HIPAA Safe Harbor guidelines.

The Personalized Therapeutic Optimization Dataset is a semi-synthetic benchmark derived from real-world omics data and simulated drug-response labels. Gene expression and methylation profiles were obtained from The Cancer Genome Atlas (TCGA, https://www.cancer.gov/tcga) and the Gene Expression Omnibus (GEO, GSE25066, https://www.ncbi.nlm.nih.gov/geo/). Drug-gene interaction information was sourced from DrugBank and DGIdb. Treatment response labels were simulated using biologically-informed heuristics inspired by DeepSynergy and MOLI pipelines. The simulation protocol and metadata specifications will be released upon publication.

The Prognostic AI Framework Dataset utilizes the HiRID dataset (https://physionet.org/content/hirid/1.1.1/), a high-resolution ICU dataset from Bern University Hospital, containing physiological measurements, medication records, and clinical interventions collected between 2008 and 2016. In-hospital mortality and time-to-discharge are used as survival targets. Cohort selection includes adult ICU patients with complete admission-discharge episodes. All data are anonymized and made available under the PhysioNet Credentialed Health Data Use Agreement.

The Multimodal Chronic Disease Diagnosis Dataset is built from diverse open-access sources: tabular clinical data from eICU-CRD, free-text clinical notes from MIMIC-III NoteEvents, retinal imaging from the EyePACS dataset used in the Kaggle Diabetic Retinopathy Detection Challenge (https://www.kaggle.com/c/diabetic-retinopathy-detection), and sensor simulation data aligned to the WESAD wearable stress dataset (https://ubicomp.eti.uni-siegen.de/home/datasets/). Labeling is based on ICD-10 codes cross-referenced with discharge diagnoses and physician annotations.

All datasets are either publicly available or simulated using reproducible pipelines. No protected health information (PHI) is included. All patient identifiers were removed or masked in compliance with HIPAA or GDPR regulations. The Chronic Disease Multimodal Analytics Dataset (Wang et al., 2025) consists of a large-scale collection of electronic health records (EHR), imaging data, and laboratory results from patients with chronic diseases such as diabetes, cardiovascular conditions, and respiratory disorders. This dataset integrates structured and unstructured modalities, enabling researchers to explore correlations between patient history, diagnostic imaging, and lab values. Each entry includes a timeline of patient visits, medications, and imaging studies, allowing for temporal modeling of disease progression. The dataset contains over 150,000 patient records collected across multiple healthcare institutions over a 10-year period. It supports both supervised and self-supervised learning paradigms. The diversity and longitudinal nature of the data make it particularly suitable for developing robust multimodal learning algorithms aimed at chronic disease prediction, progression modeling, and treatment outcome forecasting. All data are de-identified and aligned to a standardized schema to support reproducibility and cross-site generalization research.

The Personalized Therapeutic Optimization Dataset (Duda-sikuła and Kurpas, 2024) focuses on individual-level treatment strategies by combining genomic data, clinical interventions and therapeutic outcomes. It includes multi-omics information such as gene expression, methylation profiles, and proteomics, paired with detailed treatment regimens and response assessments. The dataset includes data from over 20,000 patients across oncology, rheumatology, and metabolic diseases. Each patient profile includes pre-treatment biomarker panels, administered drugs, dosage schedules, and longitudinal outcome tracking including toxicity, response metrics, and quality-of-life indices. This dataset is designed to facilitate personalized medicine research by enabling the training of machine learning models that recommend tailored interventions based on patient-specific biological and clinical signatures. The dataset adheres to FAIR data principles and includes curated subsets for benchmarking therapeutic optimization models.

The Prognostic AI Framework Dataset (Haque et al., 2023) is designed for the development and evaluation of machine learning frameworks for prognostic tasks across multiple disease types. It integrates multi-modal sources including time-series vitals, diagnostic codes, imaging sequences, and physician notes. The dataset comprises over 100,000 labeled instances from hospital admission to discharge, with outcomes such as readmission, mortality, and complication rates. It enables training of multi-task models that predict long-term and short-term clinical risks. The rich temporal structure and heterogeneous modalities present a challenging benchmark for predictive modeling. The dataset includes gold-standard labels validated by clinical experts, and is stratified by demographics to support fairness and generalization analyses. Benchmark splits and evaluation metrics are provided to ensure reproducibility of results across institutions.

The Multimodal Chronic Disease Diagnosis Dataset (Bhoi et al., 2022) aims to support diagnosis of chronic conditions through the fusion of clinical text, lab results, wearable sensor data, and imaging. The dataset includes 75,000 cases, each annotated with ICD-10 diagnostic codes and multi-modal raw data. Clinical text is extracted from physician notes and discharge summaries, while sensor data include continuous glucose monitoring and heart rate variability. Imaging modalities include MRI, CT, and retinal fundus scans. The multimodal integration enables researchers to evaluate various fusion strategies, including early, intermediate, and late fusion architectures. This dataset supports both single-label and multi-label classification tasks and includes baseline performance from existing SOTA models. Data augmentation utilities and pre-processing pipelines are provided to facilitate experimentation.

It is important to clarify that all datasets used in this study are publicly available, de-identified, and either real-world but anonymized (MIMIC-IV, eICU-CRD) or synthetically simulated from clinical patterns (for controlled policy evaluation). No private or identifiable human data were collected or used. Therefore, while the proposed framework demonstrates strong potential for chronic disease management, its current validation remains at the retrospective, non-interventional level. Claims regarding clinical utility should be interpreted as preliminary and foundational, rather than indicative of deployment readiness.

### 4.2 Experimental details

All experiments were implemented using PyTorch 2.0 and conducted on an NVIDIA A100 GPU cluster with 80 GB memory per GPU. Each training job utilized 8 GPUs in a distributed data-parallel setup. Mixed precision training with NVIDIA Apex was adopted to accelerate computation and reduce memory consumption. The training code was optimized for reproducibility using fixed random seeds, deterministic operations, and logging of all relevant hyperparameters and configurations.

For data preprocessing, all text data was tokenized using a domain-adapted version of the BioClinicalBERT tokenizer, while imaging data (MRI, CT, retinal scans) were resized to 224×224 and normalized using modality-specific mean and standard deviation. Sensor data and time-series inputs were resampled to a uniform sampling frequency and standardized per patient. Missing values in structured data were imputed using forward fill for time-series and median values for static features. Categorical variables were encoded using learned embeddings, while numerical features were normalized using z-score transformation.

Our multimodal backbone followed a dual-stream transformer architecture. Clinical text and tabular inputs were processed via a BERT-based encoder with 12 layers, 768 hidden dimensions, and 12 attention heads. Imaging inputs were processed using a vision transformer (ViT-B/16) pretrained on ImageNet, and sensor data was modeled using a temporal convolutional network (TCN) with dilated convolutions. The outputs from all modality-specific encoders were projected into a shared latent space of dimension 512 and fused using a cross-modal attention module with 4 heads and 3 layers. During training, modality dropout was applied with a probability of 0.1 to enhance robustness to missing modalities.

For classification tasks, a fully connected head with a sigmoid or softmax output (depending on whether the task was multi-label or single-label) was used. The loss function was a weighted sum of binary cross-entropy and focal loss to address class imbalance. For regression tasks (therapeutic response prediction), we used mean squared error loss. Optimization was performed using AdamW with weight decay of 0.01 and initial learning rate of $3\times 10^{-5}$ , which was scheduled using a linear warmup over the first 10% of training steps followed by cosine decay. Each model was trained for 50 epochs with early stopping based on validation AUROC or RMSE.

For evaluation, we used stratified 5-fold cross-validation with patient-level splits to ensure independence across folds. Metrics reported include Area Under the Receiver Operating Characteristic Curve (AUROC), Area Under the Precision-Recall Curve (AUPRC), accuracy, F1-score, and RMSE depending on task type. Statistical significance of performance differences was assessed using paired t-tests with p<0.05 considered significant. For robustness analysis, we tested model performance under modality ablation (removing one modality at inference) and under simulated noise injection in structured and sensor inputs.

All baseline models and SOTA methods were re-implemented or adapted from official repositories with hyperparameters tuned on validation sets. For fairness, all models were trained and evaluated under identical data splits and computational settings.

To support reproducibility, we provide detailed descriptions of the dataset splits, input modalities, training objectives, and loss functions used in our experiments. Specific hyperparameter configurations and ablation setups are reported in the Appendix and corresponding result tables. We acknowledge the importance of transparent and reproducible research, and we are committed to releasing the full codebase, data preprocessing scripts, and configuration files upon publication. A public GitHub repository will be made available to the community following article acceptance.

### 4.3 Comparison with SOTA methods

We conducted a comprehensive comparison of our proposed PATTN + TAIR framework against established clinical machine learning baselines across three core tasks: diagnosis, prognosis, and therapeutic optimization. To ensure relevance to real-world medical settings, we replaced object detection baselines and metrics with standard clinical models and evaluation criteria.

For binary classification tasks such as identifying chronic disease subtypes or risk states, we compared our model against Logistic Regression, XGBoost, LSTM, and Transformer-EHR architectures. Performance was evaluated using AUROC, AUPRC, and sensitivity/specificity at clinically meaningful operating points—specifically fixed positive predictive value (PPV) and negative predictive value (NPV). As shown in Table 1, our approach outperforms all baselines, achieving higher AUROC and AUPRC, while maintaining better sensitivity and specificity across thresholds. This demonstrates that the proposed method not only captures nuanced multimodal signals but also aligns with real-world diagnostic criteria.

**TABLE 1:** Performance on Diagnosis Tasks (Binary Classification). Results are reported as mean ± standard deviation over 5 runs.

Model	AUROC	AUPRC	Sensitivity @ PPV = 0.8	Specificity @ NPV = 0.9
Logistic regression	0.812 ± 0.011	0.548 ± 0.012	0.672 ± 0.017	0.701 ± 0.018
XGBoost	0.856 ± 0.009	0.612 ± 0.010	0.735 ± 0.014	0.763 ± 0.015
LSTM	0.871 ± 0.008	0.638 ± 0.011	0.744 ± 0.013	0.777 ± 0.012
Transformer-EHR	0.884 ± 0.006	0.659 ± 0.008	0.762 ± 0.010	0.794 ± 0.011
Ours (PATTN + TAIR)	**0.902** ± **0.004**	**0.683** ± **0.007**	**0.785** ± **0.010**	**0.821** ± **0.009**

For time-to-event prediction, we evaluated the concordance index (C-index), integrated Brier score (IBS), and standard Brier score across models including Cox Proportional Hazards, DeepSurv, and Transformer-based survival networks. Our method achieved the highest C-index and lowest IBS/Brier score (Table 2), indicating superior accuracy in forecasting disease progression and patient outcomes. These improvements are particularly notable in multimodal and longitudinal datasets, where patient trajectories are non-linear and complex. The personalized tensor-based representation of our model is especially effective in modeling heterogeneous disease evolution over time.

**TABLE 2:** Prognostic Performance (Time-to-Event Analysis). All metrics are reported as mean ± standard deviation over 5 runs.

Model	C-Index	IBS ( ↓ )	Brier score ( ↓ )
CoxPH	0.702 ± 0.010	0.201 ± 0.007	0.188 ± 0.008
DeepSurv	0.734 ± 0.008	0.183 ± 0.006	0.171 ± 0.007
Transformer-EHR (Surv)	0.755 ± 0.006	0.167 ± 0.005	0.159 ± 0.006
Ours (PATTN)	**0.777** ± **0.004**	**0.152** ± **0.004**	**0.141** ± **0.005**

To evaluate our adaptive policy learning mechanism, we compared PATTN + TAIR with three baseline policy models: Deep Q-Networks (DQN), Proximal Policy Optimization (PPO), and Bayesian Reinforcement Learning (BRL). We measured average cumulative reward, AUROC for treatment response classification, calibration error (ECE), and clinical utility using decision curve analysis (DCA). Results in Table 3 indicate that our method outperforms existing approaches across all metrics, particularly in improving policy calibration and treatment net benefit. This validates the effectiveness of our trajectory-aligned intervention recalibration strategy in adapting to evolving patient states and optimizing personalized care plans.

**TABLE 3:** Personalized Treatment Optimization (Policy Evaluation). All values are averaged over 5 runs and reported as mean ± standard deviation.

Policy model	Avg. Cumulative reward	AUROC (Response)	Calibration (ECE ↓ )	DCA net benefit
DQN	0.128 ± 0.010	0.782 ± 0.009	0.061 ± 0.005	0.094 ± 0.008
PPO	0.145 ± 0.011	0.793 ± 0.008	0.058 ± 0.006	0.101 ± 0.007
BRL	0.139 ± 0.009	0.781 ± 0.010	0.052 ± 0.004	0.109 ± 0.006
Ours (PATTN + TAIR)	**0.168** ± **0.007**	**0.817** ± **0.006**	**0.043** ± **0.003**	**0.127** ± **0.005**

To further assess the generalization capability of our framework, we conducted two additional validation protocols beyond the standard patient-level cross-validation. First, we implemented a time-aware evaluation, where training was performed on earlier time windows and testing on temporally later patient records. This setup mimics real-world deployment scenarios and assesses the model’s temporal robustness. Second, we conducted an external validation experiment by holding out data from one clinical site entirely as an unseen test set (site-held-out testing). The performance under both validation strategies is reported in Table 4.

**TABLE 4:** Performance under different validation settings. Results are reported as mean ± standard deviation over 5 repeated runs.

Validation setting	AUROC ↑	AUPRC ↑	Accuracy ↑	Drop from Std. CV
Standard 5-fold CV (Patient-level)	0.902 ± 0.004	0.683 ± 0.007	91.03% ± 0.24%	–
Time-aware CV (Early → late)	0.887 ± 0.006	0.661 ± 0.008	89.42% ± 0.31%	−1.61%
Site-held-out test (External validation)	0.864 ± 0.007	0.632 ± 0.009	87.38% ± 0.36%	−3.65%

Compared to standard 5-fold cross-validation, the AUROC dropped by 1.61% in the time-aware setting and 3.65% under site-held-out testing, confirming the model’s resilience while highlighting domain shift challenges in real-world deployment. To prevent information leakage, we strictly ensured that: (1) no patient records overlapped across splits; (2) imaging series were de-duplicated across folds; (3) clinical notes were assigned to single folds without temporal leakage; and (4) site and time labels were properly isolated during split generation. These practices were essential to preserve evaluation integrity and align with real-world usage scenarios.

To further ensure statistical rigor and reproducibility, we conducted all experiments with five independent random seeds and report results as mean ± standard deviation. This applies to all tasks including diagnosis (Table 1), prognosis (Table 2), treatment policy optimization (Table 3), and validation under real-world settings (Table 4). For each metric, such as AUROC, AUPRC, calibration, and DCA net benefit, we computed variability to assess consistency and generalizability across runs. The observed performance gains are statistically stable and demonstrate the robustness of our framework. All results follow standard reporting practices for biomedical machine learning studies, addressing reproducibility and evaluation transparency.

### 4.4 Ablation study

To assess the contributions of the key components in our model, we conducted an ablation study across four datasets: Chronic Disease Multimodal Analytics, Personalized Therapeutic Optimization, Prognostic AI Framework, and Multimodal Chronic Disease Diagnosis. Three ablated variants were defined: w/o Patient-Adaptive Transition Tensor Network (PATTN), w/o Trajectory-Aligned Intervention Recalibration (TAIR), and w/o Multimodal Tensor-Based Transition Dynamics (MTTD). Each component was removed independently while keeping the rest of the architecture unchanged. The results, presented in Tables 5, 6, demonstrate consistent performance degradation across all datasets and evaluation metrics when any component is removed.

**TABLE 5:** Ablation study results on our model across chronic disease multimodal analytics and personalized therapeutic optimization datasets.

Model	Chronic disease multimodal analytics				Personalized therapeutic optimization			
	Accuracy	Precision	Recall	mAP	Accuracy	Precision	Recall	mAP
W/o PATTN	87.03 ± 0.03	84.51 ± 0.03	83.12 ± 0.02	81.07 ± 0.02	89.16 ± 0.03	86.02 ± 0.02	85.24 ± 0.03	83.17 ± 0.02
W/o TAIR	88.61 ± 0.02	86.38 ± 0.02	84.22 ± 0.02	82.15 ± 0.03	90.25 ± 0.02	88.07 ± 0.03	86.10 ± 0.02	84.59 ± 0.03
W/o MTTD	86.84 ± 0.03	83.90 ± 0.03	82.94 ± 0.03	80.43 ± 0.02	88.73 ± 0.03	85.61 ± 0.02	84.50 ± 0.03	82.06 ± 0.03
Ours	**89.74** ± **0.02**	**87.55** ± **0.02**	**86.43** ± **0.02**	**84.69** ± **0.02**	**91.03** ± **0.02**	**89.28** ± **0.02**	**87.96** ± **0.02**	**86.11** ± **0.02**

**TABLE 6:** Ablation study results on our model across prognostic AI framework and multimodal chronic disease diagnosis datasets.

Model	Prognostic AI framework dataset				Multimodal chronic disease diagnosis dataset			
	Accuracy	Precision	Recall	mAP	Accuracy	Precision	Recall	mAP
W/o PATTN	88.16 ± 0.03	85.44 ± 0.03	84.33 ± 0.02	80.19 ± 0.02	89.84 ± 0.03	87.01 ± 0.02	85.25 ± 0.03	82.55 ± 0.03
W/o TAIR	89.03 ± 0.02	86.97 ± 0.02	85.76 ± 0.02	82.10 ± 0.03	91.12 ± 0.02	88.63 ± 0.02	86.87 ± 0.02	84.71 ± 0.02
W/o MTTD	87.45 ± 0.03	84.21 ± 0.03	83.70 ± 0.03	79.77 ± 0.02	89.01 ± 0.03	86.14 ± 0.03	84.92 ± 0.03	81.89 ± 0.03
Ours	**91.26** ± **0.02**	**89.44** ± **0.02**	**87.39** ± **0.02**	**85.13** ± **0.02**	**92.57** ± **0.02**	**90.75** ± **0.02**	**89.01** ± **0.02**	**86.60** ± **0.02**

The absence of PATTN leads to significant drops in accuracy and mAP, particularly in datasets involving complex temporal dynamics, such as the Multimodal Chronic Disease Diagnosis dataset, where mAP decreases by 3.56%. This highlights the importance of patient-specific temporal modeling in capturing disease progression. Similarly, removing TAIR results in reduced precision and recall, as seen in the Prognostic AI Framework dataset, where recall drops from 87.39% to 85.76%. This underscores the role of trajectory alignment in ensuring adaptive recalibration of treatment strategies. The removal of MTTD primarily affects datasets with heterogeneous modalities, such as Chronic Disease Multimodal Analytics, where accuracy decreases by 2.71%, indicating the necessity of tensor-based transition dynamics for effective multimodal integration.

These results validate the complementary roles of PATTN, TAIR, and MTTD in achieving robust performance across diverse multimodal medical datasets. The high sensitivity of performance to the removal of each component confirms the architectural innovations’ effectiveness in addressing the challenges of multimodal healthcare data.

To address the reviewer’s request for greater transparency and reproducibility, we expanded our ablation study to investigate several key hyperparameters and robustness conditions in our framework. The results are summarized in Table 7. First, we varied the tensor decomposition rank in the PATTN module from 4 to 32 and found that a moderate rank of 16 offered the best trade-off between performance and runtime. Increasing the temporal planning horizon H improved predictive accuracy up to H=6 , beyond which marginal gains were negligible.

**TABLE 7:** Extended ablation study on model hyperparameters and robustness.

Ablation variable	Setting (best in bold)	AUROC ↑	Accuracy ↑
Tensor rank	4/8/**16**/32	**0.902** ± **0.003**	**91.0%** ± **0.2%**
Horizon H	2/4/**6**/8	**0.902** ± **0.002**	**91.0%** ± **0.1%**
Reward weight λ	0.1/0.5/**1.0**/2.0	**0.902** ± **0.003**	-
KL coef β	0.01/0.05/**0.1**/0.5	**0.902** ± **0.002**	-
Modality dropout rate	0.0/**0.1**/0.3	**0.902** ± **0.002**	**91.0%** ± **0.1%**
Missing modality (text)	Removed/kept	**0.902** ± **0.002**	**91.0%** ± **0.1%**
Missing modality (Imaging)	Removed/kept	**0.902** ± **0.002**	**91.0%** ± **0.1%**

We also evaluated the sensitivity of TAIR to the reward weighting coefficient λ and the KL divergence regularization term β . Moderate values ( λ=1.0 , β=0.1 ) provided stable learning and optimal performance. Further, we studied the impact of the modality dropout rate used during training to simulate missing modalities. A moderate dropout rate of 0.1 improved robustness without significant accuracy loss.

To assess real-world readiness, we tested the model under missing-modality scenarios by removing clinical text and imaging data at inference time. While performance decreased slightly, the model remained resilient, demonstrating strong generalization under partial modality availability. Finally, we measured the average runtime per test sample (including inference and recalibration steps), which remained below 0.5 s/sample under all settings, validating the framework’s practicality for near real-time clinical decision support.

We conduct extensive ablation studies to assess the individual contributions of key architectural components, including PATTN, TAIR, and the multimodal tensor decomposition module (MTTD). As shown in Tables 5, 6, removing any of these components results in a consistent performance drop across multiple datasets and tasks, confirming their necessity for accurate representation learning and temporal modeling. Furthermore, we evaluate the impact of various hyperparameters and robustness settings, such as tensor rank, prediction horizon, KL regularization, and missing modality conditions. The results in Table 7 demonstrate that our model maintains stable performance under different configurations, validating its generalizability and resilience. These findings provide strong empirical grounding for the design choices in our framework.

## 5 Clinical deployment feasibility and robustness

To bridge the gap between research models and real-world clinical utility, we consider several critical factors in the practical deployment of our proposed framework, including computational efficiency, robustness to multimodal data imperfections, and clinical usability.

### 5.1 Computational efficiency

While our framework achieves strong predictive and personalization performance, it introduces non-trivial computational complexity due to tensorized transition modeling and real-time policy recalibration. In our experiments, average inference time per patient was below 0.5 s on an NVIDIA A100 GPU, including multimodal encoding and trajectory forecasting. For deployment in constrained environments (hospitals, edge devices), model compression strategies—such as low-rank tensor approximation, quantization, and weight pruning—can substantially reduce resource demands. Additionally, recalibration frequency in TAIR can be dynamically adjusted based on patient acuity, and batch inference or trajectory caching may further reduce latency. Future work includes exploring lightweight transformer backbones and hardware-aware architecture design to ensure scalability.

### 5.2 Robustness to missing and noisy modalities

Multimodal health data in clinical practice are often incomplete or corrupted, necessitating robust handling of uncertainty. Our model incorporates modality dropout during training, forcing the network to learn representations that are tolerant to partial modality absence. Moreover, shared latent embeddings across modalities allow the model to compensate for missing inputs via learned correlations (between structured EHR and imaging features). Future enhancements may involve uncertainty-aware modeling (Bayesian latent states), self-supervised modality pretraining, and reliability-weighted attention mechanisms to downweigh noisy channels.

### 5.3 Interpretability and workflow integration

Clinical adoption hinges on the interpretability and usability of AI models. Our framework supports interpretability in several ways: the latent state representations are temporally smooth and aligned with clinical concepts (disease progression phases), and patient-specific policies can be visualized and audited. The shared basis structure in both transition and emission models provides insight into which prototypical dynamics dominate a patient’s trajectory. Moreover, the modular design of our system allows integration into existing hospital IT infrastructure. For instance, our model can operate on EHR pipelines, imaging PACS systems, and real-time sensor APIs with minimal modification. Finally, the policy recalibration loop can be deployed in a clinician-in-the-loop setting to support rather than replace decision-making, maintaining trust and control.

## 6 Ethics statement

This study did not involve any prospective experiments on human participants. All data used in this research were obtained from publicly available, de-identified sources, including MIMIC-IV, eICU-CRD, CheXpert, TCGA, GEO, HiRID, and other open-access datasets. These datasets have been reviewed and approved for research use by their respective data custodians and institutional review boards, and are distributed under appropriate data use agreements.

Access to MIMIC-IV, eICU-CRD, and HiRID was granted through PhysioNet’s credentialed data access process, which requires completion of human subjects research training and compliance with the PhysioNet Data Use Agreement. TCGA and GEO datasets are open to the research community under NIH guidelines for secondary use of genomic and clinical data. The EyePACS dataset used in the Diabetic Retinopathy Detection Challenge was fully de-identified prior to public release by the organizers.

As no identifiable private health information (PHI) was used, and no patient-level interventions were conducted, this work is exempt from institutional IRB review. All simulated outcomes or treatment responses described in this study were generated using rule-based or model-based inference mechanisms over retrospective, anonymized data. Privacy, data security, and ethical standards were strictly followed in accordance with HIPAA and GDPR regulations where applicable.

## 7 Conclusions and future work

This study, we aimed to address the limitations of conventional clinical decision-making systems in managing chronic diseases, which often rely on static, population-level models that inadequately capture patient-specific variability and the complex, evolving nature of chronic conditions. To overcome these challenges, we proposed a novel AI-based computational framework that utilizes multimodal big data for personalized diagnosis, prognosis, and therapeutic optimization. Our framework is anchored by the Patient-Adaptive Transition Tensor Network (PATTN), a tensorized dynamical model that captures individualized disease evolution through latent state representations and higher-order temporal dependencies. Complementing this is the Trajectory-Aligned Intervention Recalibration (TAIR) module, which enables adaptive, real-time refinement of treatment policies by aligning predicted and observed patient trajectories. Experiments conducted on large-scale, multimodal datasets demonstrated that our framework significantly improves outcome prediction, personalization of interventions, and alignment of health trajectories, confirming its potential in real-world chronic care settings.

Despite the promising results, our framework has two key limitations. First, while PATTN effectively captures complex patterns, its computational overhead can be substantial, particularly in resource-constrained clinical environments. Future work should focus on optimizing model efficiency and exploring approximate inference techniques to enable broader deployment. Second, the system’s performance is highly dependent on the quality and completeness of multimodal data, which can vary across healthcare institutions. This calls for future research into robust learning techniques that can handle missing or noisy data without sacrificing accuracy. These directions will be critical for translating our approach into scalable, equitable, and clinically-integrated chronic disease management solutions.

## Data Availability

The original contributions presented in the study are included in the article/supplementary material, further inquiries can be directed to the corresponding author.
